# Viral nervous necrosis resistance in gilthead sea bream (*Sparus aurata*) at the larval stage: heritability and accuracy of genomic prediction with different training and testing settings

**DOI:** 10.1186/s12711-023-00796-0

**Published:** 2023-04-03

**Authors:** Sara Faggion, Paolo Carnier, Rafaella Franch, Massimiliano Babbucci, Francesco Pascoli, Giulia Dalla Rovere, Massimo Caggiano, Hervé Chavanne, Anna Toffan, Luca Bargelloni

**Affiliations:** 1 Department of Comparative Biomedicine and Food Science, University of Padova, Viale dell’Università, 16, 35020 Legnaro, PD Italy; 2 Division of Comparative Biomedical Sciences, OIE Reference Centre for Viral Encephalopathy and Retinopathy, Istituto Zooprofilattico Sperimentale delle Venezie (IZSVe), Padova, Italy; 3 Panittica Italia Società Agricola S.R.L., Strada del Procaccio, 72016 Torre Canne di Fasano, Italy

## Abstract

**Background:**

The gilthead sea bream (*Sparus aurata*) has long been considered resistant to viral nervous necrosis (VNN), until recently, when significant mortalities caused by a reassortant nervous necrosis virus (NNV) strain were reported. Selective breeding to enhance resistance against NNV might be a preventive action. In this study, 972 sea bream larvae were subjected to a NNV challenge test and the symptomatology was recorded. All the experimental fish and their parents were genotyped using a genome-wide single nucleotide polymorphism (SNP) array consisting of over 26,000 markers.

**Results:**

Estimates of pedigree-based and genomic heritabilities of VNN symptomatology were consistent with each other (0.21, highest posterior density interval at 95% (HPD95%): 0.1–0.4; 0.19, HPD95%: 0.1–0.3, respectively). The genome-wide association study suggested one genomic region, i.e., in linkage group (LG) 23 that might be involved in sea bream VNN resistance, although it was far from the genome-wide significance threshold. The accuracies (*r*) of the predicted estimated breeding values (EBV) provided by three Bayesian genomic regression models (Bayes B, Bayes C, and Ridge Regression) were consistent and on average were equal to 0.90 when assessed in a set of cross-validation (CV) procedures. When genomic relationships between training and testing sets were minimized, accuracy decreased greatly (*r* = 0.53 for a validation based on genomic clustering, *r* = 0.12 for a validation based on a leave-one-family-out approach focused on the parents of the challenged fish). Classification of the phenotype using the genomic predictions of the phenotype or using the genomic predictions of the pedigree-based, all data included, EBV as classifiers was moderately accurate (area under the ROC curve 0.60 and 0.66, respectively).

**Conclusions:**

The estimate of the heritability for VNN symptomatology indicates that it is feasible to implement selective breeding programs for increased resistance to VNN of sea bream larvae/juveniles. Exploiting genomic information offers the opportunity of developing prediction tools for VNN resistance, and genomic models can be trained on EBV using all data or phenotypes, with minimal differences in classification performance of the trait phenotype. In a long-term view, the weakening of the genomic ties between animals in the training and test sets leads to decreased genomic prediction accuracies, thus periodical update of the reference population with new data is mandatory.

## Background

Viral nervous necrosis (VNN), known also as viral encephalopathy and retinopathy (VER), is one of the most important diseases in aquaculture. VNN is endemic in the Mediterranean area [1] and the occurrence of the disease has been widely reported in both wild and farmed fish [2]. The pathogenesis of VNN consists in the vacuolation and necrosis of the nervous cells of the brain, spinal cord and retina [3] followed by clinical signs of neurological damages (abnormal swimming patterns, lethargy, skin darkening, and loss of appetite) [4].

The causative agent of VNN is Nodavirus [or nervous necrosis virus (NNV)], a non-enveloped icosahedral virus of the Nodaviridae family, genus Betanodavirus. In fish, four Betanodavirus genotypes have been detected according to a partial nucleotide sequence of the coat protein gene: striped jack nervous necrosis virus (SJNNV, the first isolated strain), tiger puffer nervous necrosis virus (TPNNV), barfin flounder nervous necrosis virus (BFNNV) and red-spotted grouper nervous necrosis virus (RGNNV) [5].

Gilthead sea bream (*Sparus aurata*) has long been considered not susceptible to VNN and a potential asymptomatic vector of the infection [6, 7]. After several mass mortality outbreaks observed in Mediterranean sea bream hatcheries [8–10], it was discovered that the NNV reassortant strain RGNNV/SJNNV possesses a particular tropism to sea bream and poses a new and unexpected threat to the sea bream aquaculture industry. Larval stages are the most susceptible to NNV, with rather severe outcomes (100% mortality rates) [9, 10]. The outcome of the infection seems to be less predictable in juveniles, and to depend on age and environmental conditions; however, mortality rates can still be moderate to high (10 to 100%) [9, 11]. During natural outbreaks in sea bream hatcheries, infected larvae showed a clearly recognizable symptomatology, implying clinical and behavioural signs such as lethargy, apathy, anorexia, abnormal swimming patterns, loss of balance and swim bladder hyperinflation [8–10]. Those clinical and behavioural signs were reported to always precede the death [8–10], and thus can be used as indicator traits of VNN mortality in sea bream.

Vaccines or alternative therapeutics against NNV infection are not available; and anyway, the protection of the larval stages by vaccination is precluded by the immaturity of the immune system. In sea bream, well-developed anterior kidney, thymus and spleen are observed at 1, 22–29 and 12 d post-hatching, respectively, but lymphocytes do not appear before 47–54 d post-hatching [12].

The genetic improvement of individual resistance against the reassortant NNV strain is thus a major goal in sea bream breeding. In other commercially important species (European sea bass, Asian sea bass and Atlantic cod), significant additive genetic variation for VNN resistance has been reported [13–15], which is promising for sea bream. Along with a long-term control of the disease and a cumulative and permanent increase in resistance over generations [16], selective breeding for enhanced resistance offers the opportunity to reduce disease outbreaks within offspring batches, and to enhance the sustainability and profitability of aquaculture production [17, 18].

Phenotyping for disease resistance based on challenge trials is difficult, due to the complexity of experimental infections, and limited to the relatives of breeding candidates due to biosecurity reasons. In this context, the implementation of genomic selection procedures is of great interest and is made feasible by the recent availability of high-density marker arrays for sea bream [19]. Genomic selection relies on genotyping and phenotyping, in challenge trials, a reference population consisting of full- and half-sibs of the breeding candidates. Genotypes and phenotypes of the reference population are used to estimate allele substitution effects for a dense panel of genetic markers, which are exploited in the prediction of genomic estimated breeding values (GEBV). Such estimated effects are validated in a “testing” population and then used in the routine prediction of the genetic merit of future breeding candidates, which need to be genotyped only [20].

Testing the consistency of prediction equations in a long-term view is of great importance: in commercial breeding, genomic models are first trained using a reference population consisting of full- and half-sibs of the candidate breeders. Random k-fold CV have been demonstrated to provide overly optimistic genomic prediction accuracies [21–25], thus alternative strategies to assess the accuracy of prediction equations should consider the decrease in the genomic ties between animals in training and testing sets (i.e., the animals to be predicted are distant relatives of the animals included in the reference population). For livestock species, the proportion of close relatives in training and testing sets of CV is well-known to affect the accuracy of genomic predictions both for traits with high and low heritability [22, 26, 27].

To date, in gilthead sea bream, neither the genetic variation in nor the genomic prediction accuracy for VNN resistance have been investigated. Hence, the aims of this study were to estimate the genetic and genomic parameters of VNN resistance in challenged gilthead sea bream larvae, to estimate the effects of single nucleotide polymorphisms (SNPs) on the VNN resistance phenotype through a genome-wide association study (GWAS) and to assess the accuracy of genomic predictions of the trait or EBV using a novel SNP array for sea bream. The accuracy of genomic prediction models was assessed using both random CV and two validation settings that mimic a reduction of the genomic relatedness between training and testing sets: a CV based on genomic clustering and a leave-one-family-out validation focused on the parents of the challenged fish.

## Methods

### Production of the experimental fish and NNV challenge test

The experimental fish were produced in a commercial hatchery (Panittica Italia Società Agricola srl, Torre Canne di Fasano, Brindisi, Italy) in three independent full-factorial mating designs (10 sires × 12 dams, 10 sires × 7 dams, 9 sires × 6 dams). At 16 d post-hatching, nearly 1300 sea bream larvae, NNV-free tested, were transferred to the Istituto Zooprofilattico Sperimentale delle Venezie (IZSVe, Legnaro, Padova). Larvae were distributed into one close-system 250-L tank filled with artificial saltwater at a salinity of 35–37‰ (i.e., UV and carbon filtered tap water with artificial balanced salt, Instant Ocean- Aquarium System), a temperature of 19 ± 2 °C and oxygen 6–8 ppm, and exposed to an artificial photoperiod of 8 h of light and 16 h of darkness. After five days of acclimation, larvae were infected by immersion using the reassortant betanodavirus strain (RGNNV/SJNNV) isolated from animals belonging to a fish farm during a disease outbreak (VNNV/S.aurata/Farm1/461-1/Nov2014) [8]. Two hundred and fifty mL of viral suspension (10^7.80^ TCID_50_/mL) (TCID_50_ for median tissue culture infectious dose 50%) was added to the tank. The final infectious titre, obtained by back titration of the water, was 10^5.45^ TCID_50_/mL. Larvae were exposed to the same amount of virus at the same time. A few hours after distributing the viral suspension, the water in the tank was discarded and replaced with clean water. Fish were checked three times a day to identify individuals showing typical clinical signs of VNN (abnormal swimming patterns, lethargy). As soon as a symptomatic larva was detected, it was sacrificed and stored in 96% ethanol for subsequent genomic DNA extraction. The experiment ended 14 d post-challenge and non-symptomatic larvae were sacrificed and stored in 96% ethanol. Overall, 1229 symptomatic and non-symptomatic larvae were collected.

The experimental infection protocol was evaluated by the Istituto Zooprofilattico Sperimentale delle Venezie (IZSVe) Animal Welfare Body and Ethics Committee (Opinion CE.IZSVE.3/2016 of 24/10/216) and approved by the Italian Ministry of Health (Law decree 101/2017-PR of 02/02/2017).

### Genomic DNA extraction, genotyping, parentage assignment and survival analysis

Genomic DNA (gDNA) was extracted from the whole larva using a modified salting-out protocol described by Pardo et al. [28]. gDNA concentration was quantified using the Qubit dsDNA BR Assay Kit (Invitrogen – Life Technologies) and gDNA integrity was assessed by visualization on a 1% TAE agarose gel stained with SYBR® Safe DNA Gel Stain (Invitrogen – Thermo Fisher Scientific). gDNA was successfully extracted from 1044 of the 1229 larvae. gDNA from 54 parental tissue samples (fins) was extracted with the Invisorb Spin Tissue Mini Kit (Invitek Molecular Berlin).

Genotyping of the experimental fish and their parents was performed at IdentiGEN (Ireland) using the MedFish SNP array [19] which contains approximately 30K SNPs for the gilthead sea bream. Nine hundred and seventy-four fish and all their parents (54) were successfully genotyped. SNP quality control and genotype calling from the intensity files were performed using the Axiom™ Analysis Suite software v. 5.1.1.1 (Thermo Fisher Scientific) with default parameter values for diploid species (call rate > 97%, dish quality control (QC) > 0.82; QC is a metric provided by ThermoFisher generated by measuring signals at multiple sites on the genome known to be invariable across individuals), resulting in 27,706 available SNPs per fish. SNPs with a minor allele frequency (MAF) lower than 1%, a missing genotype in more than 15% of the individuals or that deviated from the expected Hardy–Weinberg equilibrium frequencies (*p* < 0.001) were discarded. Missing genotypes were imputed using the FImpute software [29], resulting in a dataset of 26,591 SNP genotypes per fish.

The APIS/R [30] software package was used to assign parentage. It uses the observed distributions of average Mendelian transmission probabilities to set assignment thresholds, and enables the estimation of the proportion of offspring with missing parental genotypes. For all APIS runs, parameters were set at default values, with the exception of the error rate, which was set to 5%. Parentage assignment to a unique parental pair was achieved for all the experimental fish. In total, 160 full-sibs families were identified as being generated by 23 dams and 29 sires, with the number of offspring per family ranging from 1 to 55. The number of offspring per sire ranged from 1 to 125, while the number of offspring per dam ranged from 1 to 339. After removing the family with a size of 1, 972 individuals from 22 dams and 28 sires were retained for the subsequent analyses.

The Kaplan–Meier product-limit survival curve of the challenged fish was estimated using survival/R [31].

### Additive genetic variance and pedigree-based heritability

Estimation of the variance components, parameters, and prediction of breeding values were based on the solution of a threshold (probit) animal model. In such a model, the observed binary phenotypes for VNN symptomatology (y = 0 for the asymptomatic animals and y = 1 for the symptomatic ones) were assumed to depend on the value of an unobservable and normally distributed latent variable $\uplambda$ (liability) such that:

$$\mathrm{P}\left(\mathrm{y}=0|\mathrm{x}\right)=\mathrm{P}\left(\uplambda \le {\uplambda }_{\mathrm{t}}|\mathrm{x}\right),$$

and $\mathrm{P}\left(\mathrm{y}=1|\mathrm{x}\right)=\mathrm{P}\left(\uplambda >{\uplambda }_{\mathrm{t}}|\mathrm{x}\right),$ where ${\uplambda }_{\mathrm{t}}$ is a critical cut-off value or threshold and $\mathrm{x}$ is a set of explanatory variables. Using the cumulative standard normal density function as a link function, such probabilities can be obtained as follows:

$$\mathrm{P}\left(\uplambda \le {\uplambda }_{\mathrm{t}}|\mathrm{x}\right)=\Phi \left({\uplambda }_{\mathrm{t}}-\uplambda |\mathrm{x}\right),\mathrm{ P}\left(\uplambda >{\uplambda }_{\mathrm{t}}|\mathrm{x}\right)=1-\Phi \left({\uplambda }_{\mathrm{t}}-\uplambda |\mathrm{x}\right),$$

where $\Phi \left(\cdot \right)$ denotes the cumulative standard normal density function (probit function).

The model used to interpret the variation in the liability was:

1 $${\boldsymbol{\uplambda}}= {\boldsymbol{\upmu}}+ \mathbf{W}\mathbf{u}+\mathbf{e},$$

where ${\boldsymbol{\uplambda}}$ is a vector of liabilities, ${\boldsymbol{\upmu}}$ is a constant vector corresponding to model intercept, $\mathbf{u}$ is a vector of unknown random animal additive genetic effects, $\mathbf{W}$ is an incidence matrix relating ${\boldsymbol{\uplambda}}$ to $\mathbf{u}$, $\mathbf{e}$ is a vector of random residuals. Additive genetic effects and the residuals were assumed to be distributed as $\mathrm{N}\left(0,\mathbf{A}{\upsigma }_{a}^{2}\right)$ and $\mathrm{N}\left(0,{\mathbf{I}\upsigma }_{e}^{2}\right)$, respectively, where $\mathrm{N}()$ indicates a normal probability density function, $\mathbf{A}$ is the numerator relationship matrix, ${\upsigma }_{a}^{2}$ is the additive genetic variance, $\mathbf{I}$ is an identity matrix of appropriate order and ${\upsigma }_{e}^{2}$ is the residual variance.

Marginal posterior distributions of the genetic variance in VNN symptomatology were estimated using a Bayesian approach employing Monte-Carlo Markov chain (MCMC) and the Gibbs sampler, as implemented in the software TM [32]. A single Gibbs chain of 1,000,000 samples was generated, the first 1000 were discarded as burn-in and from the remaining 999,000 samples, we saved one from every 100. The convergence of each Markov chain was checked using the Geweke convergence diagnostic [33].

Heritability of VNN symptomatology was estimated as:

2 $${\mathrm{h}}_{p}^{2}= \frac{{\upsigma }_{a}^{2}}{{\upsigma }_{p}^{2}},$$

where ${\upsigma }_{a}^{2}$ is the additive genetic component of the variance and ${\upsigma }_{p}^{2}$ is the phenotypic variance (i.e., ${\upsigma }_{p}^{2}={\upsigma }_{a}^{2}+{\upsigma }_{e}^{2}$). The median of the estimated marginal posterior distribution was used a point estimate of the heritability. The 95% highest posterior density intervals (HPD95%) and the probability for ${\mathrm{h}}_{p}^{2}$ to be greater than 0.2 (arbitrary critical threshold) were obtained from the estimated posterior density of ${\mathrm{h}}_{p}^{2}$ using the boa/R software [34].

### Prediction of breeding values

Estimated breeding values for VNN symptomatology were obtained using Model (1), using all available information (i.e., the observed phenotype of the individual and those of its full- and half-sibs). Variance components used to solve the model were those obtained in the previous analysis. A Bayesian approach employing MCMC and Gibbs sampling methods was implemented using the software TM [32].

### Genomic variance and heritability

Genomic variance and genomic heritability for VNN symptomatology were estimated with Bayesian procedures as implemented in BGLR/R [35] and following the methodology described by de Los Campos et al. [36]. Briefly, given ${\upsigma }_{a}^{2}= {\upsigma }_{g}^{2}+ {\upsigma }_{\overline{g} }^{2}$, where ${\upsigma }_{a}^{2}$ is the additive genetic variance of the trait, ${\upsigma }_{g}^{2}$ represents the amount of additive genetic variance captured by a linear regression on available marker genotypes (i.e., the genomic variance) and ${\upsigma }_{\overline{g} }^{2}$ is the “missing” additive genetic variance, the genomic heritability is defined as the proportion of variance in phenotypes explained by the regression on marker genotypes:

3 $${\mathrm{h}}_{g }^{2}={\mathrm{h}}_{p}^{2}\frac{{\upsigma }_{g}^{2}}{{\upsigma }_{a}^{2}}=\frac{{\upsigma }_{g}^{2}}{{\upsigma }_{p}^{2}},$$

where ${\mathrm{h}}_{p}^{2}$ is the pedigree-based heritability of the trait and $\frac{{\upsigma }_{g}^{2}}{{\upsigma }_{a}^{2}}$ is the proportion of additive genetic variance explained by a linear regression on marker genotypes. Hence, the upper limit of the genomic heritability is the pedigree-based heritability, that we assumed to be properly estimated using Model (1) and Eq. (2).

Three regression models were implemented: Bayes B, Bayes C [37] and Bayesian Ridge Regression [38]. A major difference between the models is represented by the prior density used for the marker genotype effects; further details can be found in Pérez and de Los Campos [35]. All models included an intercept and the effects of the animal genotypes at 26,591 SNPs, coded as the number of copies of the minor allele (0, 1 or 2). Features of the estimated marginal posterior density of genomic heritability were obtained through the same procedure as that described for the pedigree-based heritability.

### Genome-wide association study

A GWAS was performed to test the association between the VNN symptomatology phenotype and SNP genotypes (26,591 SNPs) using gaston/R [39] following a mixed logistic regression model:

4 $${\boldsymbol{\upeta}}=\upmu +\mathbf{W}\mathbf{u}+\mathbf{z}\mathrm{\alpha },$$

where ${\boldsymbol{\upeta}}$ is a vector of logits as $\mathrm{log}\left[\frac{{\uppi }_{\mathrm{i}}}{1- {\uppi }_{\mathrm{i}}}\right]$, ${\uppi }_{\mathrm{i}}$ is the probability that the phenotype is 1 (symptomatic) for animal $\mathrm{i}$, $\upmu$ is the model intercept, $\mathbf{u}$ is an unknown vector of random animal additive genetic effects assumed to be $\mathrm{N}\left(0, \mathbf{G}{\upsigma }_{a}^{2}\right)$ where $\mathrm{N}\left(\right)$ indicates a normal probability density function, $\mathbf{G}$ is the genomic relationship matrix and ${\upsigma }_{a}^{2}$ is the estimated additive genetic variance, $\mathbf{W}$ is an incidence matrix relating the vector of logits ${\boldsymbol{\upeta}}$ to $\mathbf{u}$, $\mathrm{\alpha }$ is the unknown allele substitution effect of a single SNP, $\mathbf{z}$ is a vector of marker genotypes, coded as the number of copies of the minor allele, relating ${\boldsymbol{\upeta}}$ to $\mathrm{\alpha }$. The genome-wide significance threshold was set to p-value = 0.05/p, where p is the number of SNPs considered in the analysis. Hence, to address the problem of multiple testing, a Bonferroni correction was applied to the significance threshold. Manhattan plots of GWAS were obtained with the R package ggplot2 [40].

### Genomic predictions

Genotypes at 26,591 SNPs were used as predictors of the phenotype or pseudo-phenotypes (EBV) for VNN symptomatology. Three Bayesian regression models were fitted to the data using BGLR/R [35]: Bayes B, Bayes C and Bayesian Ridge Regression. Bayesian regression models were used because they simultaneously fit a larger number of markers than the number of observations available for the analysis (the well-known “small n, large p” problem), by combining phenotypic information with priors for marker effects and variance components. Markers are fitted as random effects, accounting for most of the genetic variance. The general form of such models was:

5 $$\mathbf{y}= {\boldsymbol{\upmu}}+ \mathbf{X}{\boldsymbol{\upbeta}}+\mathbf{e},$$

where $\mathbf{y}$ is a vector of pseudo-phenotypes (EBV) or, in the case of binary phenotype (VNN symptomatology), corresponds to a vector of liabilities, ${\boldsymbol{\upmu}}$ is the vector of the constant term or model intercept, $\mathbf{X}$ is a $\mathrm{n}\times \mathrm{p}$ matrix of SNP genotypes ($\mathrm{n}$ is the number of animals, $\mathrm{p}$ is the number of markers), coded as the number of copies of the minor allele (0, 1 or 2), ${\boldsymbol{\upbeta}}$ is a $\mathrm{p}\times 1$ vector of regression coefficients (SNP effects) to be estimated, and $\mathbf{e}$ is a vector of random residuals. The residuals were assumed to be $\mathbf{e}\sim \mathrm{N}\left(0,{\mathbf{I}\upsigma }_{e}^{2}\right)$, where $\mathrm{N}\left(\right)$ indicates a normal probability density function, $\mathbf{I}$ is an identity matrix of appropriate order and ${\upsigma }_{e}^{2}$ is the residual variance.

Each Bayesian analysis was carried out generating a single Gibbs chain of 800,000 samples, with a burn-in of 100,000 iterations and a thinning interval of 50 samples.

In the analysis of the phenotype for VNN symptomatology, the probit function was used as a function to link the probability of each category (0 = asymptomatic, 1 = symptomatic) to the linear predictor, whereas a linear model was used for pseudo-phenotypes (EBV); the residual variance was set to 1.

### Model performance for the prediction of EBV

Prediction performance of the Bayesian models was assessed in three CV strategies: (a) a fourfold random CV, (b) a fourfold CV based on genomic clustering (K-means clustering), and (c) a leave-one-family-out (LOFO) validation, focused on the parents of the challenged animals. These strategies were used to mimic three genomic selection scenarios, where the genomic prediction equations are obtained by training models with information from a reference population that could be more closely (random CV) or more distantly (K-means clustering and LOFO) related to the animals to be predicted.

In the first strategy (a), the dataset consisting of EBV that were obtained according to the methodology described above and Model (1), was randomly split into four equally-sized data segments. Seventy-five percent of the data (training set) were used to obtain the solutions for allele substitution effects. The pseudo-phenotypes of the remaining 25% of the data (test set) were masked and predicted from the genotypes using solutions of the Bayesian models obtained in the analysis of the training set. The analysis was repeated four times; predictions for each test set were saved and, at the end, aggregated with those of the other test sets. Prediction accuracy was measured as the Pearson product-moment correlation between EBV and genomic-predicted values of EBV. To assess prediction bias, the regression coefficient (slope, β) of the EBV on the genomic-predicted EBV was also calculated, with slopes equal to 1 indicating absence of bias.

The accuracy in the estimation of the additive genetic value, which depends on the accuracy of EBV and on the accuracy of genomic-predicted EBV, was calculated as in [41]:

6 $$r=\sqrt{{(corr(\mathrm{EBV},\mathrm{ genomic\,predicted\,EBV}))}^{2}\cdot reliability\left(\mathrm{EBV}\right)},$$

where ${(corr(\mathrm{EBV},\mathrm{ genomic\,predicted\,EBV})}^{2}$ is the squared Pearson product-moment correlation between EBV and genomic-predicted values of EBV; reliabilities of EBV were calculated, for each individual, as ${r}_{\mathrm{EBV}}^{2}=1-(\frac{\mathrm{PEV}}{{\sigma }_{a}^{2}})$, where $\mathrm{PEV}$ is the prediction error variance, and ${\sigma }_{a}^{2}$ is the additive genetic variance.

In the second strategy (b), the K-means method clustered the animals according to distance of genomic relationships, reducing the relatedness between training and testing populations while maximizing the relatedness within each cluster. K-means clustering was performed using the function kmeans (stats/R) [42], which implements the Hartigan and Wong algorithm [43].

Clusters were used one at a time as a test set. As in (a), pseudo-phenotypes (EBV obtained according to the methodology described above and Model (1)) of the test set were masked and predicted from the genotypes using solutions of the Bayesian models obtained in the analysis of the training set consisting of the other three clusters. The predictions were saved and, at the end of the procedure, aggregated with those of the other clusters to compute, as a measure of prediction accuracy, the Pearson product-moment correlation between EBV and genomic-predicted EBV; prediction bias was assessed as the regression coefficient (slope, β) of the EBV on the genomic-predicted EBV.

In the third procedure (c), the EBV obtained according to the methodology described above and Model (1), were retrieved for the parents of the experimentally-infected fish. Solutions for allele substitution effects were obtained by training the models on the EBV of all animals except the offspring of the sire (or the dam) used as a test set (just one parent in the test set). The EBV of the parent in the test set was then predicted from its genotypes by making use of the solutions of Bayesian models obtained in the analysis of the training set. This was replicated as many times as the number of parents. The prediction and EBV for each parent were saved and at the end of the LOFO, aggregated with those of the other parents to compute the Pearson product-moment correlation between the EBV and the genomic-predicted EBV; prediction bias was assessed as the regression coefficient (slope, β) of the EBV on the genomic-predicted EBV.

### Model performance for the classification of the phenotype for VNN symptomatology

Different classifiers of the phenotype for VNN symptomatology were tested, including the EBV, the genomic predictions of the phenotype and of the EBV provided by Bayesian models. Classification performance was assessed in a set of five independently generated fourfold CV. For each CV, three metrics were used to evaluate the performance for the classification of the phenotype for VNN symptomatology: the area under the receiving operating characteristics (ROC) curve (AUC), the classification accuracy (ACC) computed as (true positives + true negatives)/total number of samples, and the Matthews correlation coefficient (MCC). All the metrics were computed using the package ROCR/R [44].

## Results

### Nervous necrosis virus challenge test

**Fig. 1:**
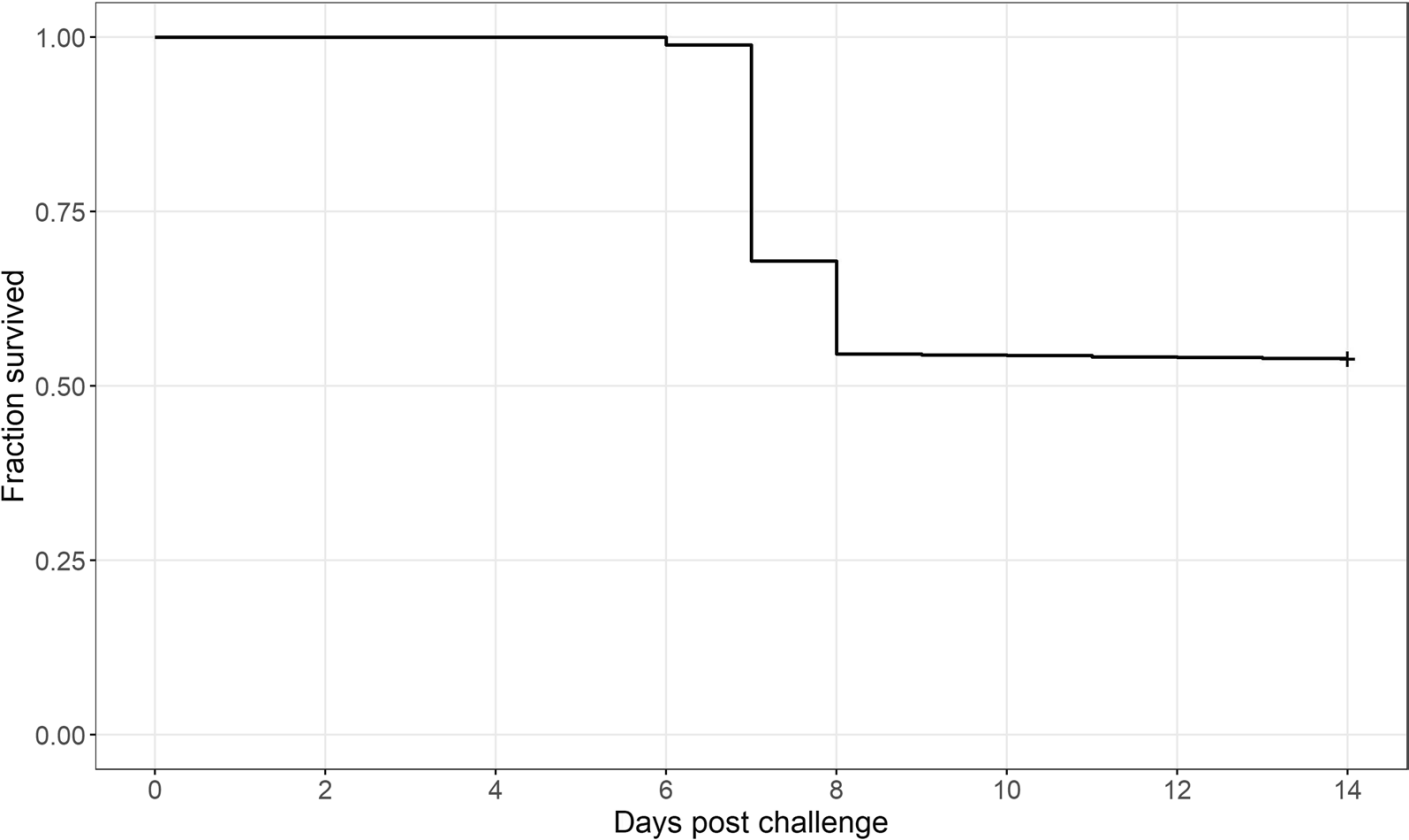
Kaplan–Meier product-limit curve for survival of fish during the challenge test with NNV

The Kaplan–Meier product-limit survival curve for the challenged fish is reported in Fig. 1. Mortality reached a peak at day 7 post-challenge. In total, 566 symptomatic larvae were identified between days 6 and 13 post-challenge. At the end of the experiment, 663 animals were classified as survivors and were sacrificed (overall survival rate: 53.9%).

### Additive genetic variance and pedigree-based heritability

The median of the marginal posterior density for the additive genetic (${\sigma }_{a}^{2}$) and phenotypic variance (${\sigma }_{p}^{2}$) for VNN symptomatology was equal to 0.263 and 1.263, respectively. Viral nervous necrosis symptomatology had a moderate heritability (${\mathrm{h}}_{p}^{2}$ = 0.208 on the underlying scale), with a probability of 57.2% for the estimate to be higher than the critical value of 0.2. The estimated 95% highest posterior density interval for heritability was narrow, with values between 0.077 and 0.366.

### Genomic variance and genomic heritability

Point estimates and features of the marginal posterior densities for variance components and genomic heritability for VNN symptomatology are in Table 1. The estimates provided by the three Bayesian models were similar; Bayes C provided the highest heritability estimate (${\mathrm{h}}_{g}^{2}$ = 0.195), while the estimates provided by Bayes B and Bayes Ridge Regression were 1.03 and 0.7 percentage points lower, respectively. The estimated genomic heritability averaged over models (${\mathrm{h}}_{g}^{2}$ = 0.188) was similar to the pedigree-based heritability, and the probability for the estimate to be higher than 0.2 ranged from 38.6% (Bayes B) to 50.2% (Bayes C). The width of the HPD95% intervals for the estimated genomic heritability was approximately half that for ${\mathrm{h}}_{p}^{2}$, which indicates that the uncertainty in the estimation process of the genomic heritability was lower.

**Table 1:** Estimates of the genomic variance (${{{\upsigma}}}_{{{g}}}^{2}$) and heritability (${{h}}_{{{g}}}^{2}$) for VNN symptomatology, 95% highest posterior density interval (HPD95%) for ${{h}}_{{{g}}}^{2}$ and probability for ${{h}}_{{{g}}}^{2}$ to be greater than 0.2 obtained with three Bayesian regression models

Model	${\upsigma }_{g}^{2}$	${\mathrm{h}}_{g}^{2}$	HPD95%	P (${\mathrm{h}}_{g}^{2}$≥ 0.2)
Bayes B	0.222	0.182	0.106, 0.262	0.386
Bayes C	0.242	0.195	0.118, 0.283	0.502
Bayes Ridge Regression	0.232	0.188	0.120, 0.259	0.434

### Genome-wide association study

**Fig. 2:**
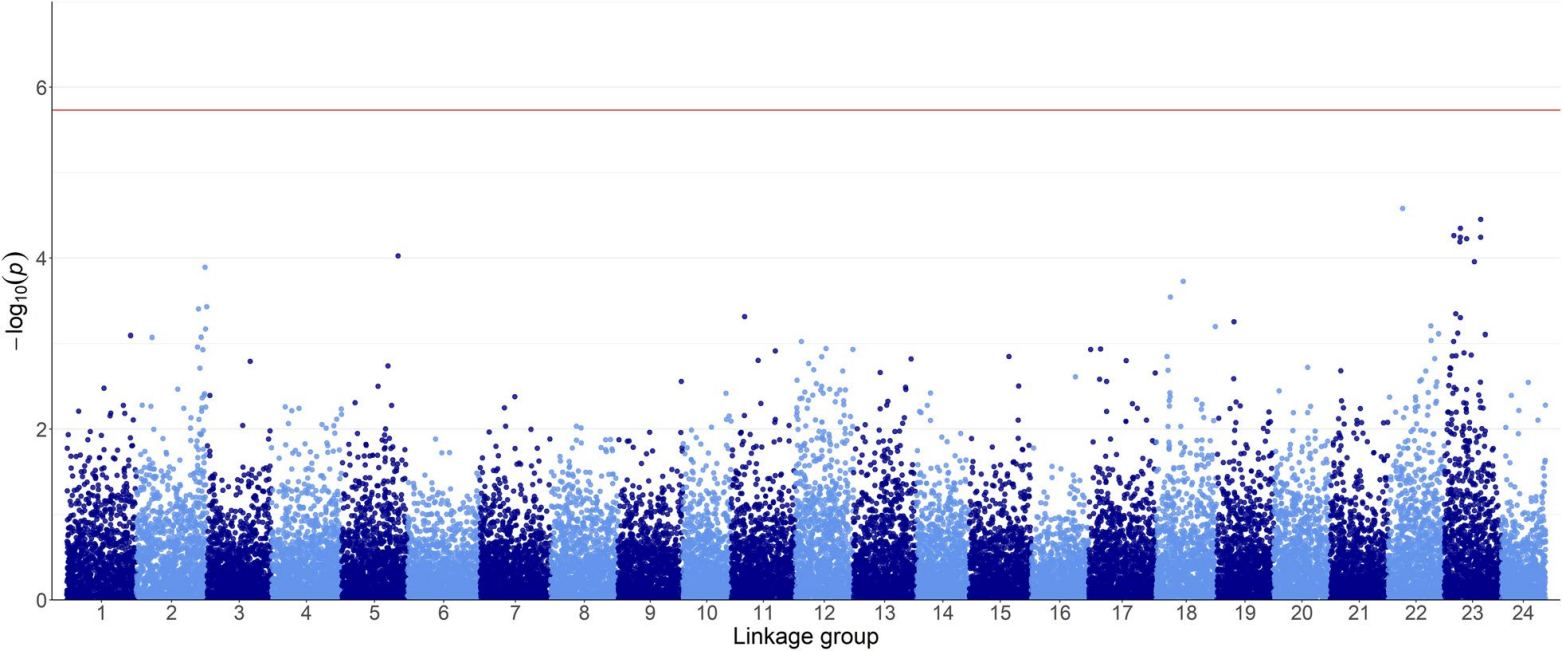
Genome-wide association plots between genotypes at 26,591 SNPs and VNN symptomatology. The red line indicates the genome-wide significance threshold p-value = 0.05/p (where p is the total number of SNPs) after Bonferroni correction and − log_10_ transformation (5.73)

In the GWAS, the genome-wide significance threshold was set to 5.73 (after Bonferroni correction and -log_10_ transformation). The GWAS did not identify any SNP exceeding the genome-wide significance level (Fig. 2); SNPs located on linkage groups 22 and 23 are those that are closest to the significance level.

### Average genomic relatedness within and between training/testing sets

In random CV, the dataset was split into four equally-sized subsets (Table 2). The genomic relatedness both within group and between one group and the others was comparable across groups. K-means clustering produced four groups composed of an unbalanced number of animals (Table 2). The genomic relatedness within each group was maximized (values ranging from 0.04 to 0.26), whereas the relatedness between one group and the others was minimized (values ranging from − 0.06 to − 0.02; Table 2). The average genomic relatedness within the group of parents was 0.015, whereas between challenged fish and the population of parents was -0.002.

**Table 2:** Number of animals (N) in each group, average genomic relatedness (standard deviation SD) within group (g_ij_w_) and average genomic relatedness (SD) between one group and the others (g_ij_b_), for each cross-validation strategy

Cross-validation strategy	Group	N	g_ij_w_	g_ij_b_
Random	1	243	− 0.001 (0.113)	− 0.001 (0.114)
	2	243	− 0.001 (0.116)	− 0.001 (0.115)
	3	243	− 0.001 (0.114)	− 0.001 (0.115)
	4	243	− 0.001 (0.118)	− 0.001 (0.116)
K-means clustering	1	336	0.125 (0.099)	− 0.064 (0.070)
	2	177	0.239 (0.114)	− 0.054 (0.073)
	3	326	0.039 (0.100)	− 0.024 (0.069)
	4	133	0.258 (0.098)	− 0.043 (0.077)
Leave-one-family-out	Parents	54	0.015 (0.088)	− 0.002 (0.109)

For the leave-one-family-out validation, g_ij_w_ refers to the group of parents, whereas g_ij_b_ refers to the parents and the challenged fish

### Model performance for genomic prediction of the estimated breeding values

In the random CV procedure, genomic predictions of the EBV for VNN symptomatology were highly accurate and unbiased, with a Pearson product-moment correlation equal to 0.90 and β coefficients (slope of the regression of the observed on the predicted EBV) equal to 1.01 (Table 3). The comparison between Bayesian genomic models showed that their performance for prediction was similar. The accuracy of the prediction of the true additive genetic value, which depends on the accuracy of EBV and on the accuracy of genomic-predicted EBV, ranged from 0.44 to 0.51 and was, on average, 0.46.

**Table 3:** Accuracy of prediction of EBV for VNN symptomatology and β coefficient provided by the Bayesian models in three cross-validation strategies (random, K-means clustering and leave-one-family-out focused on the parents of the challenged fish)

Cross-validation strategy	Model	Accuracy	β
Random	Bayes B	0.903	1.010
	Bayes C	0.903	1.010
	Bayes Ridge Regression	0.903	1.010
K-means clustering	Bayes B	0.525	1.935
	Bayes C	0.530	1.956
	Bayes Ridge Regression	0.529	1.952
Leave-one-family-out	Bayes B	0.119	0.587
	Bayes C	0.126	0.620
	Bayes Ridge Regression	0.127	0.625

*EBV* breeding value estimated using the phenotypic information of the animal and of its full- and half-sibs

*Accuracy* Pearson product-moment correlation between EBV and genomic-predicted EBV

*β* slope of the regression of the observed on the predicted EBV

The prediction accuracy decreased to 0.53 when the genomic relatedness between training and testing sets was minimized (K-means clustering) and dropped remarkably (0.12) when the most penalizing validation (LOFO), which is able to break down the family linkage disequilibrium due to the experimental design, was applied (Table 3).

The three Bayesian models exhibited similar prediction accuracies and similar β coefficients, both in the K-means clustering validation and in the LOFO validation (Table 3).

### Model performance for the genomic classification of VNN symptomatology

When used as classifiers of VNN symptomatology, breeding values estimated from the observed phenotype of the individual and those of its full- and half-sibs (EBV) exhibited an AUC of 0.905, an ACC of 0.864 and a MCC of 0.730. The classification of the observed symptomatology based on the genomic-predicted phenotype resulted in moderate performances for all methods, showing AUC between 0.581 and 0.601, ACC between 0.569 and 0.583, and MCC between 0.175 and 0.178 (Table 4). The genomic-predicted EBV used as a classifier provided an added value, increasing the metrics to 0.657 (AUC), 0.617 (ACC) and 0.237 (MCC). The results were consistent across Bayesian models, with slightly greater metrics for Ridge Regression.

**Table 4:** Average metrics (SD) for classification of VNN symptomatology based on genomic predictions of phenotype or of breeding values as classifiers

Classifier	Method	Metric		
		AUC	ACC	MCC
Genomic-predicted phenotype	Bayes B	0.581 (0.017)	0.569 (0.016)	0.175 (0.024)
	Bayes C	0.597 (0.013)	0.581 (0.013)	0.176 (0.023)
	Bayes Ridge Regression	0.601 (0.013)	0.583 (0.009)	0.178 (0.022)
Genomic-predicted EBV	Bayes B	0.657 (0.002)	0.617 (0.006)	0.237 (0.008)
	Bayes C	0.657 (0.002)	0.617 (0.006)	0.237 (0.008)
	Bayes Ridge Regression	0.657 (0.002)	0.616 (0.005)	0.236 (0.008)

*EBV* breeding values estimated using the phenotypic information of the animal and of its full- and half-sibs

*AUC* area under the ROC curve, *ACC* accuracy as (true positives + true negatives)/number of samples, *MCC* Matthews correlation coefficient

## Discussion

The recently discovered susceptibility of gilthead sea bream to a NNV reassortant strain has posed a new and unexpected threat to the sea bream Mediterranean aquaculture [9, 10]. Selective breeding for enhanced genetic resistance of sea bream against VNN aims at protecting larval stages, whose immune system is still in development [12], and may exploit evidence on the same pathology obtained for European sea bass, Asian sea bass and Atlantic cod [13–15] or on other diseases for other species [17]. Incorporation of genomic information in selection procedures is of great interest for complex traits such as disease resistance and is encouraged by the development of high-density marker arrays [19]. To date, in sea bream, the genetic basis of VNN resistance has not been investigated yet. Moreover, studies based on experimental infections in early developmental stages are scarce, particularly those with samples of size suitable to estimate variance components and genetic parameters.

In this study, the experimental trial was conducted on very young fish (21 d post-hatching) and of small size compared to previous studies (150–200 d post-hatching, 10–20 g) [45–48]. In spite of the young age and small size of the fish, clinical signs were clearly recognizable and guaranteed a reliable recording of the symptomatology. As reported in case studies focused on natural outbreaks in sea bream hatcheries, the clinical signs preceding the death in larvae aged 17–35 d post-hatching were, as commonly described for VNN, lethargy, apathy, anorexia, abnormal swimming, loss of balance and swim bladder hyperinflation [8–10].

Due to the small size of the fish, the experimental infection was carried out by immersion which, when compared to infection by injection, leads to outcomes which depend on the same defence mechanisms as those activated in natural disease outbreaks. The limited size of the experimental tank guaranteed an equal level of exposure to the virus for all animals, which is important when assessment of across-family differences in resistance is the key aspect of the study.

The mortality rate in our study (46.1%) was comparable to that reported in sea bream of similar age experiencing natural infections (40%) [10]. Previous studies on natural outbreaks reported significantly higher mortality rates (> 80%) [8, 9], which may result from different environmental conditions (water temperature and density) and from inherent genetic differences across populations in terms of VNN resistance as detected, for example, in sea bass [46].

Frequently, investigations on the genetic variation in disease resistance are focused only on mortality assessed in challenge tests, where fish are classified as dead or survived after a controlled viral, bacterial or parasitic infection. In future studies, it could be worth disentangling the genetic variation of the different mechanisms implied in survival, as already performed in pacific white shrimp (*Penaeus vannamei*) for susceptibility and endurance to taura syndrome [49] and in Atlantic cod (*Gadus morhua*) for susceptibility and endurance to VNN and vibriosis [45]. Although the traits under consideration were characterized by different pedigree-based heritability estimates (${\mathrm{h}}_{p}^{2}$ = 0.33–0.91 for susceptibility and ${\mathrm{h}}_{p}^{2}$ = 0.02–0.12 for endurance), those studies provided encouraging results that could motivate further investigations on different traits which have a role in survival, rather than focusing only on mortality.

The heritability estimated for VNN symptomatology (${\mathrm{h}}_{p}^{2}$ = 0.21 on the underlying scale) was in the range of estimates obtained for VNN resistance in European sea bass (0.14–0.38) [13, 46–48, 50] and Asian sea bass (*Lates calcarifer*; 0.25) [14], but lower than estimates obtained for VNN resistance in Atlantic cod (0.43–0.91) [15, 45, 51, 52]. It must be noted that all the cited experiments were performed on fish of different size and age compared to our experimental animals and, with the exception of the study of Palaiokostas et al. [47], they also used a different method of infection (injection), which may lead to differences in the estimated heritability.

The genomic heritability estimate (${\mathrm{h}}_{g}^{2}$ = 0.19) was consistent with the pedigree-based heritability estimate, which indicates that SNP effects could pick up all the variation due to additive genetic effects. In addition, the HPD95% interval for the genomic heritability was approximately half as large as that of the pedigree-based heritability (0.115–0.268 vs. 0.077–0.366), which indicates that ${\mathrm{h}}_{g}^{2}$ was estimated with less uncertainty and that genomic inference is more accurate than the pedigree-based one. The genomic heritability estimate is anyway lower than that obtained by Palaiokostas et al. [47] and Vela-Avitúa et al. [48] in sea bass using genomic information (0.40–0.43), but, since in those studies the experimental animals had undergone several full cycles of selection, the linkage disequilibrium derived from selective breeding could be a reason for such an overrating of the additive genetic variance.

The weak statistical power of single-marker GWAS [53] led, in our case, to unsatisfactory results in terms of detection of genomic regions significantly associated with VNN resistance in gilthead sea bream. One genomic region located in LG23 was suggested to be involved in sea bream VNN resistance, as it is the region closest to the significance threshold. Likewise, in European sea bass a linkage group explaining 9.2% of the variance was associated with VNN resistance [48, 50]. In our study, the presence of three minor genomic regions explaining 2.6 to 2.8% of the genetic variance reinforces once again the hypothesis that disease resistance traits are complex and polygenic. Overall, the total variance explained by the top four windows was 11.73%.

At first glance, the obtained genomic prediction values seem to be encouraging in view of the practical implementation of genomic selection in sea bream to enhance the resistance to the NNV reassortant strain, but the CV strategy may affect estimates of prediction accuracy; in particular, random k-fold CV performed on close relatives from a single generation may lead to overly optimistic results, as already demonstrated by different authors both in livestock [21, 22] and aquaculture species [23–25]. The availability of data from only one generation is due to the complexity of the experiments required to record the disease resistance traits, which prevents routine phenotyping, and to the fact that in aquaculture species, unlike terrestrial species, breeding programs for disease resistance (and to complex traits in general) have only recently started and are not widely applied yet [54].

In our study, the accuracy of genomic prediction of EBV provided by random fourfold CV was high (*r* = 0.90), but, since this strategy may not be informative in the long-term due to the disruption of family-based linkage disequilibrium, two other validation strategies (K-means clustering and LOFO) were tested to mimic more realistic scenarios where the genomic ties between animals in the training and testing sets due to close relationships, as those occurring in full- and half-sib families, were weakened. As expected, both K-means clustering and LOFO accuracies decreased in comparison with that estimated in random fourfold CV. Although in the K-means clustering strategy the accuracy remained moderate, suggesting that genomic selection could be feasible in a mid-term view, in the LOFO validation strategy the inflection was marked (*r* = 0.12). Such differences across validation strategies may also be ascribed to the use of the EBV as pseudo-phenotypes. Due to the mating design and the moderate heritability of VNN symptomatology, EBV include a family component, which, in our case, represents the largest proportion of the EBV variance. The genomic prediction accuracies obtained in the CV procedures, where the animals included in the training and testing sets were randomly chosen, are expected to be high because many sibs are in both validation and training sets and family means are predicted with family information. When family data are not considered in the validation procedure, as in LOFO and, to a lower extent, in K-means clustering, the accuracy of the genomic prediction is expected to be low. Even with individual phenotypes, the difference between validation strategies would have been observed, but it would have been more limited.

Anyway, in Atlantic salmon, Fraslin et al. [25] evidenced how a systematic reduction of the relatedness between the training and testing sets resulted in decreased genomic prediction accuracies, compared to that of the random CV. When the genomic relationship between the training and testing populations was set to an extreme value of 0.3, the accuracy for sea lice resistance and body weight decreased up to -44% and -51%, respectively. In our study, the scenario was even more extreme, as the parent contribution to the challenged sample was rather unbalanced, with three dams out of 22 producing almost 67% of the individuals, and six sires out of 28 producing nearly 48% of the individuals. In the K-means and LOFO validation procedures, this resulted in weak relationships between the parent used as a test set and the animals in the training set. de Los Campos et al. [55] demonstrated how, when the data involve unrelated individuals, linkage disequilibrium spans over shorter regions and within-family disequilibrium cannot be exploited. This implies an imperfect linkage disequilibrium between markers and causal loci, which, in turn, results in very low prediction accuracy of genomic best linear unbiased prediction (GBLUP). Estimated breeding values for VNN symptomatology was the best classifier of the observed trait (AUC = 0.91), which was expected since the individual phenotypic information was included in the data used to estimate the breeding value. The classification of the observed VNN symptomatology based on the genomic prediction of the phenotype exhibited an AUC equal to 0.59, whereas genomic prediction of EBV provided a 12% increase of the classification performance (AUC = 0.66). The AUC metric for classification based on the genomic predictions of the phenotype was in the range of values reported for other species challenged with viral diseases (0.52 to 0.70 for VNN in sea bass, [47, 50]; 0.74 for koi herpesvirus disease in common carp, [56]).

The fish used in this study were produced from a breeding stock that has never undergone a selective breeding program directed to the enhancement of resistance to VNN; in this context, several steps are specific to the planning of an efficient and long-term view breeding program: (1) the creation of a breeding nucleus which develops the élite broodstock, (2) the design of breeding strategies to produce both full-sib and half-sib families, (3) the development of a high-depth pedigree database including all the information of breeding candidates and tested fish, and (4) the exploitation of genomic information, which can be combined to the aforementioned information to accurately select the breeding candidates.

## Conclusions

The estimated heritability for VNN mortality indicates that selective breeding programmes are feasible for increasing resistance to VNN of fish larvae/juveniles, which would overcome the problem of vaccination. Traditional selective breeding approaches for VNN resistance are expected to have a limited efficiency, mainly due to the low accuracy of the estimated breeding values of the breeding candidates which, for sanitary reasons, cannot be included in VNN challenge tests. The results of the GWAS suggest a complex polygenic nature for VNN resistance with many genomic regions each with a small effect on the phenotypic variation of the trait. In view of the implementation of genome-enabled selection in sea bream to enhance resistance to the NNV reassortant strain, the accuracies of the genomic predictions were high when assessed in CV. However, the application of validation procedures, which can cancel the favourable effects of family-based linkage disequilibrium that are determined by the experimental design, decreased dramatically the estimated accuracies. This raises concerns on the use of factorial mating design paired with CV as a tool to assess genomic prediction accuracies and emphasizes the need of the periodical update of the training set with new data to keep prediction accuracies at a satisfactory level in the case of populations where, like the one investigated in this study, genomic relationships across families are weak. Further studies should focus on disentangling the genetic variation in the different mechanisms that have a role in VNN survival, such as susceptibility and endurance, rather than recording only mortality of experimentally-infected animals.

## Data Availability

Data are available from the authors upon reasonable request.
